# Prevention and Alleviation of Dextran Sulfate Sodium Salt-Induced Inflammatory Bowel Disease in Mice With *Bacillus subtilis*-Fermented Milk *via* Inhibition of the Inflammatory Responses and Regulation of the Intestinal Flora

**DOI:** 10.3389/fmicb.2020.622354

**Published:** 2021-01-07

**Authors:** Xuan Zhang, Yanjun Tong, Xiaomei Lyu, Jing Wang, Yuxue Wang, Ruijin Yang

**Affiliations:** ^1^State Key Laboratory of Food Science and Technology, Jiangnan University, Wuxi, China; ^2^School of Food Science and Technology, Jiangnan University, Wuxi, China

**Keywords:** *Bacillus subtilis*, intestinal flora, functional food, inflammatory bowel diseases (IBD), probiotics

## Abstract

The pathogenesis of inflammatory bowel disease (IBD) might be related to the local inflammatory damage and the dysbacteriosis of intestinal flora. Probiotics can regulate the intestinal flora and ameliorate IBD. The probiotic *Bacillus subtilis* strain *B. subtilis* JNFE0126 was used as the starter of fermented milk. However, the therapeutic effects of *B. subtilis*-fermented milk on IBD remain to be explored. In this research, the therapeutic effect of *B. subtilis-*fermented milk on dextran sulfate sodium salt (DSS)-induced IBD mouse model was evaluated. Besides, the expression of pro-inflammatory/anti-inflammatory cytokines, the proliferation of the intestinal stem cells, and the reconstruction of the mucosa barrier were investigated. Finally, alteration of the gut microbiota was investigated by taxonomic analysis. As shown by the results, the disease activity index (DAI) of IBD was significantly decreased through oral administration of *B. subtilis* (JNFE0126)-fermented milk, and intestinal mucosa injury was attenuated. Moreover, *B. subtilis* could reduce the inflammatory response of the intestinal mucosa, induce proliferation of the intestinal stem cell, and promote reconstruction of the mucosal barrier. Furthermore, *B. subtilis* could rebalance the intestinal flora, increasing the abundance of *Bacillus*, *Alistipes*, and *Lactobacillus* while decreasing the abundance of *Escherichia* and *Bacteroides*. In conclusion, oral administration of the *B. subtilis*-fermented milk could alleviate DSS-induced IBD *via* inhibition of inflammatory response, promotion of the mucosal barrier reconstruction, and regulation of the intestinal flora.

## Introduction

Inflammatory bowel disease (IBD) is a chronic inflammatory disease of the intestines, which has emerged as a global disease. The robust epidemiologic and experimental studies have defined the role of lifestyle and microbiome on the pathogenesis of this disease (Ng et al., 2018; Ananthakrishnan et al., 2020). The pathogenesis of IBD is associated with intestinal flora imbalance and overexpression of pro-inflammatory cytokines due to autoimmune response, which results in apoptosis of epithelial cells and formation of a local ulcer. The pharmacotherapy of IBD includes the use of aminosalicylates, corticosteroids, 6-mercaptopurine, and other immunosuppressive agents (Ali Sohrabpour et al., 2010). Recently, antibodies of tumor necrosis factor (TNF) have become a novel strategy (Pugliese et al., 2017; Armuzzi et al., 2020). However, the aforementioned therapies have side effects and limitations of various degrees. In comparison with traditional therapy and monotherapy, probiotic/dietary therapy, and combinations of some therapeutic approaches might possess better patient acceptability and therapeutic effect in the prevention and curation of IBD (D’Haens et al., 2014; Durchschein et al., 2016; Jadhav et al., 2020; Mentella et al., 2020; Targownik et al., 2020).

Recent reports revealed that alteration of the gut microbiota was associated with IBD, including the change of relative abundance and the decreased diversity of the microbiota. This reduction in richness of the microbiota was seen for *Firmicutes*, *Actinobacteria*, *Bacteroidetes*, *Collinsella*, *Lactobacillus*, and *Bacillus* (Maukonen et al., 2015; Alipour et al., 2016; Sjöberg et al., 2017). To restore flora balance in the IBD, some probiotic products have been applied to regulate the intestinal flora for treatment of this disease in recent reports (Plaza-Díaz et al., 2017; Amer et al., 2018; Bian et al., 2019; Jang et al., 2019; Chen et al., 2020; Wang et al., 2020). Fermented foods, which contained edible microorganisms, have been investigated as dietary supplements for the prevention and treatment of IBD. Fermented barley and soybean showed protective function against IBD through the effects on the inflammatory reaction, tight junction protein expression, and gut microbiota composition in animals (Woo et al., 2016). Saraiva et al. (2015) reported that milk fermented with a 15-lipoxygenase-1-producing *Lactococcus lactis* alleviated the symptoms of colitis in a murine model.

*Bacillus subtilis* is a probiotic found in the Japanese traditional food natto. Recent clinical researches reported that oral intake of *B. subtilis* was effective in preventing pediatric diarrhea and reducing the duration of diarrhea (Guo et al., 2019). *B. subtilis* can secrete some bioactive substances beneficial to human health and has been applied as a probiotic in humans (Lefevre et al., 2017). Yan et al. (2020) reported that surfactin A from *B. subtilis* could inhibit inflammation and promote wound healing. Johnson et al. (2019) reported that peptidoglycan-associated cyclic lipopeptide (a surfactin) from *B. subtilis* could disrupt CoV virion integrity in the HUH7 cells and reduce infection > 10,000-fold, and its viricidal activity extended broadly to enveloped viruses, including SARS-CoV, MERS-CoV, influenza, Ebola, Zika, Nipah, chikungunya, Una, Mayaro, Dugbe, and Crimean-Congo hemorrhagic fever viruses. It was also reported that poly-γ-glutamic acid secreted from *B. subtilis* could attenuate the symptoms and histological features of IBD and reduce inflammation in an animal model of colitis (Davaatseren et al., 2013). Foligné et al. (2012) reported that spores from the *B. subtilis* strain PB6 possessed anti-inflammatory effects on the TNBS-induced colitis in mice. In addition, some functional factors in milk, such as conjugated linoleic acid (CLA) and milk polar lipids (MPLs), have been reported to possess anti-inflammatory function. CLA could ameliorate dextran sulfate sodium salt (DSS)-induced colitis in mice model (Chen et al., 2019), and MPLs could ameliorate necrotizing enterocolitis (NEC) in a rat model by inhibiting the inflammatory response (Yang et al., 2020). Lorea Baroja et al. (2007) reported that yogurt containing probiotics possessed anti-inflammatory effects in IBD patients. These reports prompt that *B. subtilis-*fermented milk might be applied as a functional food for the prevention and curation of IBD in the future.

Inflammatory bowel disease includes ulcerative colitis (UC) and Crohn’s disease (CD). CD has more serious symptoms due to extensive inflammatory injury of both the small intestinal and colonic mucosa, compared to UC which showed limited pathological area of ulcers in the colon. For investigation of the pathogenesis and therapeutic mechanisms of drugs for IBD, some IBD animal model was applied for this research. DSS can corrode the mucosa and epithelium of the digestive tract and is widely used to establish animal models of IBD by oral intake of this chemical agent. In the majority of the present reports on DSS-induced IBD models, only colonic damage was investigated, and the pathological changes in the small intestine were ignored. However, it was reported by Elsheikh et al. (2012) that mucosal injury of the DSS-induced IBD existed extensively in the small intestine and the colon, which was similar to the pathological features of CD.

In the present study, the aim of this research was to identify the biological effect of orally administered *B. subtilis*-fermented milk based on the DSS-induced IBD animal experiment. For evaluation of the prevention and therapeutic effect of *B. subtilis* on DSS-induced IBD, the disease activity index (DAI) and the histological/pathological features of small intestinal and colonic mucosa were observed. Furthermore, the expression of inflammatory cytokines, the reconstruction of the epithelium barrier, and the alteration of intestinal flora were investigated to provide the underlying mechanism by which the *B. subtilis*-fermented milk alleviates IBD.

## Materials and Methods

### Materials

Dextran sulfate sodium salt (molecular weight 36,000–50,000) was purchased from MP Biomedicals, LLC (CA, United States); antibodies, including antibodies of myeloperoxidase (MPO), TNF-α, interleukin-10 (IL-10), G-protein-coupled receptor 5 (Lgr5), caudal type homeo box transcription factor 2 (CDX2), Mucin2, zonula occludens-1 (ZO-1), villin, and β-actin, were purchased from Boster Biological Technology Co., Ltd (Wuhan, Hubei, China). Anti-goat IgG-horseradish peroxidase (HRP), anti-rabbit IgG-HRP, and anti-mouse IgG-HRP, RIPA lysis buffer, and BCA Protein Assay Kit were purchased from Boster Biological Technology Co., Ltd (Wuhan, Hubei, China); polyvinylidene difluoride (PVDF) membrane was purchased from Millipore (CA, United States); Pierce ECL Plus western blotting substrate was purchased from Thermo Fisher Scientific (MA, United States); and UHT whole milk was purchased from Mengniu Dairy (Hohhot, Inner Mongolia, China). The *B. subtilis* strain JNFE0126 was isolated from natto in our experiment.

### Preparation of the *B. subtilis*-Fermented Milk

The *B. subtilis* strain JNFE0126 was used to prepare the *B. subtilis*-fermented milk. The fermentation substrate milk was composed of the UHT whole milk and 2% sucrose (w/w). The substrate milk was heated at 95°C for 5 min. After cooling down to 40°C, the *B. subtilis* strain JNFE0126 amplified in the normal medium was inoculated to the substrate milk at a concentration of 10^6^ CFU/ml. The milk was fermented at 41°C for 8 h and a firm curd was formed. The final CFU of *B. subtilis* in the fermented milk was 6 × 10^8^ CFU/ml. The *B. subtilis*-fermented milk was stored at 4°C for no more than 24 h before usage.

### Establishment of the DSS-Induced IBD Model and the Experimental Design

In this study, all animal experimental protocols were approved by the Ethics Committee of Jiangsu University for the use of experimental animals and conformed to the Guide for the Care and Use of Laboratory Animals. Male C57BL6/J mice, 8 weeks old and weighing 22–23 g, were raised at room temperature (25 ± 2°C) and were exposed to a 12-h light/dark cycle with free access to standard rodent chow and water in the barrier facility of the Animal Center of Jiangsu University. Then, the 100 mice were divided into five groups (20/group, Table 1 shows the experimental procedures): (1) The control (normal) group was fed with standard chow and drank sterile water; (2) the *B. subtilis*-fermented milk group was fed with standard chow and drank sterile water; in addition, 300 μl of the *B. subtilis*-fermented milk was orally given by gavage twice daily for 21 days; (3) the DSS group was fed with standard chow for 21 days and drank sterile water during the first 7 days and the third 7 days. For inducing IBD, during the second 7 days, the normal drinking water was replaced by 3.5% DSS (in drinking sterile water, *ad libitum* access). In addition, 300 μl of normal saline was orally given by gavage twice daily for 21 days. (4) The DSS + milk group was fed with standard chow for 21 days and drank sterile water during the first 7 days and the third 7 days. For inducing IBD, during the second 7 days, the normal drinking water was replaced by 3.5% DSS (in drinking sterile water, *ad libitum* access). In addition, 300 μl of substrate milk was orally given by gavage twice daily for 21 days. (5) The DSS + *B. subtilis*-fermented milk (FM) group was fed with standard chow for 21 days and drank sterile water during the first 7 days and the third 7 days. For inducing IBD, during the second 7 days, the normal drinking water was replaced by 3.5% DSS (in drinking sterile water, *ad libitum* access). In addition, 300 μl of *B. subtilis*-fermented milk was orally given by gavage twice daily for 21 days for the prevention and cure of IBD. During the experimental time, the health and symptom of the animals including diarrhea and blood in fecal matter was monitored twice a day. The body weight was recorded every day. Five mice from each group were executed at 14 days (the active phase of IBD, after oral intake of DSS for 7 days), and the other 15 mice were executed at 21 days (the recovery phase of IBD, after the termination of DSS intake for 7 days) of the experiment. The intestines were taken out from the abdominal cavity, and their gross appearance was observed and then washed with phosphate-buffered saline (PBS). These intestine samples were prepared for histological observation and western blotting assay, and the intestinal contents were used for the analysis of intestinal flora in the following experiments.

**TABLE 1:** The group design of the animal experiment.

**Groups**	**0–7 days**	**8–14 days**	**15–21 days**
Normal	<————————Normal drinking water————————>		
	Standard chow		
*B. subtilis*-fermented milk	<————————Normal drinking water————————>		
	Standard chow + 300 μl *B. subtilis*-fermented milk twice daily by oral gavage		
DSS	Normal drinking water	3.5% DSS in drinking water	Normal drinking water
	Standard chow + 300 μl normal saline twice daily		
DSS + milk	Normal drinking water	3.5% DSS in drinking water	Normal drinking water
	Standard chow + 300 μl fermentation substrate milk twice daily by oral gavage		
DSS + *B. subtilis*-fermented milk	Normal drinking water	3.5% DSS in drinking water	Normal drinking water
	Standard chow + 300 μl *B. subtilis*-fermented milk twice daily by oral gavage		

### Assay for Disease Severity Evaluation

#### Disease Activity Index Assessment

The DAI was adopted to evaluate the severity of colitis (Kim et al., 2012). Body weight and the shape and consistency of stools of the animals were checked every day. The DAI scoring system was defined as follows. For weight, 0 indicated no weight loss, 1 indicated 5–10% weight loss, 2 indicated 10–15% weight loss, 3 indicated 15–20% weight loss, and 4 indicated greater than 20% weight loss. For stool, 0 indicated normal stool, 2 indicated loose stool, and 4 indicated diarrhea. For bleeding, 0 indicated no blood, 2 indicated the presence of blood, and 4 indicated gross blood. For observation of disease progression, the DAI scoring was performed daily from day 8 to day 21 of the experiment.

#### Appearance Observation of the Intestines

At 7 days after termination of DSS administration, the mice were deeply anesthetized with pentobarbital sodium (200 mg/kg, intraperitoneal injection) and then the small intestine and colon were taken out from the abdominal cavity. These samples were photographed and their appearance, including the shape integrity, surface gloss, and elasticity, was observed to assess preliminarily the severity of injury and bleeding in the intestines caused by DSS-induced IBD. The length of the colons of the animals in different groups was recorded to compare the disease severity of DSS-induced IBD among these groups.

### Histology Observation and Scoring

The samples of ileum and colon taken out from the DSS-induced IBD animal models in the active phase (on termination of DSS administration) and the recovery phase (at 7 days after termination of DSS administration) of IBD were fixed with paraformaldehyde. After dehydrated with gradient ethanol, the samples were embedded in paraffin and then sectioned (5 μm thick). The sections were stained with hematoxylin and eosin (H&E staining). In addition, the sections were stained with Alcian blue for observation of the goblet cells (which contained mucus) in the mucosa of the intestines. H&E-stained tissue sections of the small intestine and the colon were scored by a blinded observer with the following measures (Kim et al., 2012): villin/crypt architecture (normal, 0 to severe villin/crypt distortion with loss of entire villin/crypts, 3), degree of inflammatory cell infiltration (normal, 0 to dense inflammatory infiltrate, 3), muscle thickening (base of crypt sits on the muscularis mucosae, 0 to marked muscle thickening present, 3), goblet cell depletion (absent, 0 to present, 1), and crypt abscess (absent, 0 to present, 1). The histological damage score is the sum of each individual score.

### Immunohistochemical Staining

At 7 days after termination of DSS administration, all mice were sacrificed, and intestines including the ileum and colon were taken out and then fixed in 4% paraformaldehyde. After dehydrated with gradient ethanol, the samples were embedded in paraffin and then sectioned (5 μm thick) for immunohistochemical staining (IHC). For observation of the infiltration of neutrophils, the inflammatory reaction, and the cellular distribution of pro-inflammatory cytokine and anti-inflammatory cytokine in the intestinal mucosa, the antibodies against the neutrophil marker MPO, pro-inflammatory cytokine TNF-α, and anti-inflammatory cytokine IL-10 were used to stain the sections of ileum and colon of IBD animal models. Meanwhile, to observe the distribution of intestinal stem cells in the mucosa, the epithelial regeneration, and the reconstruction of the mucosal barrier after the treatment, the specific proteins, including the intestinal stem cell marker Lgr5, the goblet cell marker Mucin2, the intestinal epithelial function proteins CDX2 and villin, and tight junction protein ZO-1, were stained by IHC using the specific antibodies. In the procedures of IHC, the second antibodies were the corresponding IgG-HRP, and positive staining was shown brown *via* DAB. The negative control test of IHC was carried out *via* replacement of specific antibodies against these proteins by the normal serum of rabbit, goat, or mouse.

### Western Blotting

At 7 days after termination of DSS administration, the samples of the same long (5 cm) distal ileum and colon in the normal group, DSS + milk group, and DSS + *B. subtilis*-fermented milk group were collected and washed twice with PBS. For extraction of the tissue proteins, the samples were lysed with RIPA lysis buffer (100 mg/ml) containing the protease inhibitor cocktail on ice for 30 min and ultrasonicated at 4°C, and then centrifuged at 5,000 *g* for 5 min. Protein concentrations of the supernatant were quantified with the BCA assay kit and then mixed with equal amounts of loading buffer and denatured by heating at 100°C for 5 min. The proteins from these samples (of equal amount) were loaded and separated by SDS-PAGE on 10% polyacrylamide gels and then transferred to PVDF membranes. The membranes were blocked with blocking solution for 1 h, and then incubated with primary antibodies against target proteins, including MPO, TNF-α, IL-10, Lgr5, CDX2, Mucin2, ZO-1, villin, and β-actin (as standard control) for 12 h at 4°C. For showing of the bands of these target proteins, the membranes were incubated with secondary antibodies conjugated with HRP and visualized using a Pierce ECL Plus substrate, and then scanned with Typhoon 9400 Variable Mode Imager (Amersham Biosciences). The ratio of gray scale between the target protein band and β-actin band was calculated as the relative expression level of the target protein. Each experiment was repeated at least three times for statistical analysis (*n* = 5).

### Intestinal Flora Analysis

At 7 days after termination of DSS administration, the mice were deeply anesthetized with pentobarbital sodium (200 mg/kg, intraperitoneal injection) and the abdominal cavity was open. The total colons of five mice of each group (five groups) were taken out. The total content of the colon was washed out with PBS and stored at −80°C. Total genomic DNA was extracted from the colonic content using the E.Z.N.A. Soil DNA Kit (Omega, United States). The V3–V4 hypervariable region of the bacterial 16S rRNA gene was amplified for species classification by PCR with the following primers: the forward primer (CCTACGGGNGGCWGCAG) and the reverse primer (GACTACHVGGGTATCTAATCC). The AMPure XP beads were used to remove the free primers and primer dimer species in the amplicon product. Samples were delivered to Sangon BioTech (Shanghai, China) for library construction using universal Illumina adaptor and index. Sequencing was performed using the Illumina MiSeq system (Illumina MiSeq, United States). The sequences were clustered into operational taxonomic units of at least 97% identity. The classification of the taxonomic analysis was operated based on the mothur software package according to the standard pipeline described in the mothur website.

### Statistical Analysis

All data were presented as the mean ± standard deviation (SD). Statistical analysis was performed by SPSS 13.0 statistical software. The two-way analysis of variance (ANOVA) was used to analyze the difference between groups, and *p* < 0.05 was considered to be statistically significant.

## Results

### The Appearance of the *B. subtilis*-Fermented Milk and the Microscope Observation of the Gram Staining Sections

After 8 h of fermentation, the *B. subtilis*-fermented milk formed a firm curd with a yellowish-white color, which showed no whey separation. After stirring, the *B. subtilis*-fermented milk became viscous fluid. This fermented milk possessed the sensory properties and overall acceptability similar to traditional fermented milk product (data not shown). The gram-positive *Bacillus* in this fermented milk could be observed after gram staining, and the bacterial concentration was 6 × 10^8^ CFU/ml.

### Effect of the *B. subtilis*-Fermented Milk on Disease Severity of the DSS-Induced IBD

**FIGURE 1:**
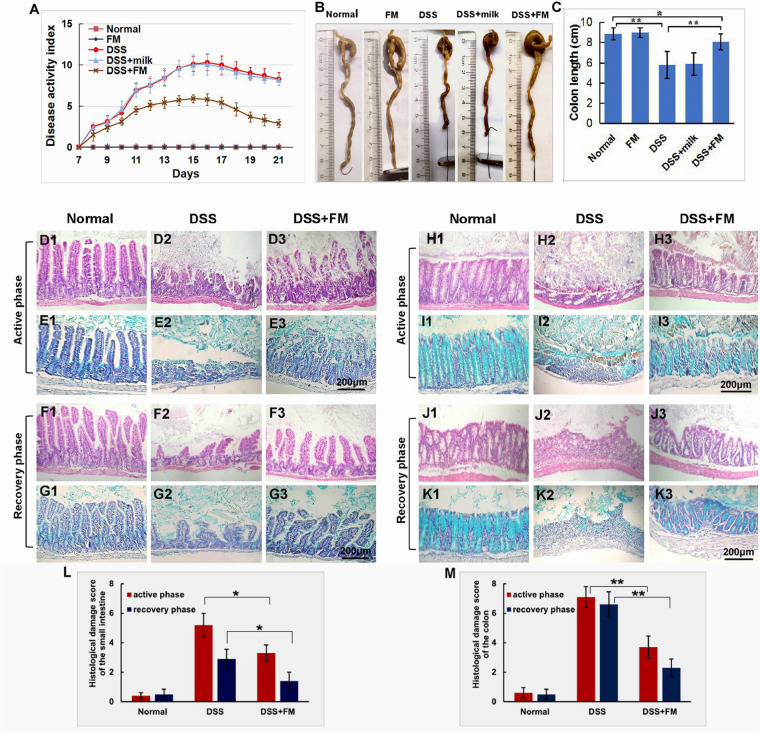
Effects of the *B. subtilis*-fermented milk on the disease severity and the pathological changes in the small intestine and the colon of the DSS-induced IBD. **(A)** The DAI dynamic changes of the DSS-induced IBD animal models. During the second 7-day period (active phase of IBD), DAI in the DSS + *B. subtilis*-fermented milk group was lower than those of the DSS group and the DSS + milk group (*n* = 15, *p* < 0.01). In the third 7-day period (recovery phase of IBD), after termination of DSS intake, DAI recovery with varying degrees was observed in the DSS group, the DSS + milk group, and the DSS + *B. subtilis*-fermented milk group. However, in the DSS + *B. subtilis*-fermented milk group, the DAI index recovered faster and was significantly lower than those of the DSS group and the DSS + milk group (*n* = 15, *p* < 0.01). The DSS + milk group showed no obvious difference from the DSS group (*n* = 15, *p* > 0.05). **(B)** The colonic appearance of the model animals at 7 days after termination of DSS administration. **(C)** Quantification analysis of the length of the colons in different groups. At 7 days after the termination of DSS intake, the colons in the DSS group and the DSS + milk group were shortened significantly compared with those in the normal group and *B. subtilis*-fermented milk group. However, in the DSS + *B. subtilis*-fermented milk, the colons were shortened only slightly and were significantly longer than those of the DSS group and the DSS + milk group. The *B. subtilis*-fermented milk group showed no obvious difference from the normal group, and the DSS + milk group showed no obvious difference from the DSS group (*n* = 15, * represents *p* < 0.05, ** represents *p* < 0.01). **(D,E)** Changes in the histological structure of the small intestinal mucosa in the active phase of the DSS-induced IBD observed with HE staining and Alcian blue staining, respectively. **(D1,E1)** The normal group: the villi in the small intestinal mucosa were finger-like, which were arranged compactly and tidily. The crypt structure was integrated among the base of villi. The surface of the villi and the crypt were covered with epithelial cells and goblet cells, with abundant mucus; **(D2,E2)** the DSS group: the necrosis and ulcer of the mucosa were observed, the villi were disintegrated, and only some residual crypts were observed; **(D3,E3)** the DSS + *B. subtilis*-fermented milk group: damage in the small intestinal mucosa was relatively slight, and the crypt structure was almost integrated. **(F,G)** Changes in the histological structure of the small intestinal mucosa in the recovery phase of the DSS-induced IBD observed with HE staining and Alcian blue staining, respectively. **(F1,G1)** The normal group: no significant change was observed in the mucosa; **(F2,G2)** the DSS group: the small intestinal mucosa was partially recovered, and the villi were short and scattered; **(F3,G3)** the DSS + *B. subtilis*-fermented milk group: the intestinal mucosa epithelium and the crypts were integrated, although the regenerative villi were shorter than those of the normal small intestines. **(H,I)** Changes in the histological structure of the colonic mucosa in the active phase of the DSS-induced IBD observed with HE staining and Alcian blue staining, respectively. **(H1,I1)** The normal group: the colonic mucus were intact; **(H2,I2)** the DSS group: the deep ulcer was observed due to the damage of the epithelium and glands; **(H3,I3)** the DSS + *B. subtilis*-fermented milk group: fewer bleeding and necrosis were observed. The damage of the colonic mucosa was relatively slight, and only local superficial ulcers were observed. A major part of the epithelium and glands was integrated in the structure, and the goblet cells were filled with abundant mucus. **(J,K)** Changes in the histological structure of the colonic mucosa in the recovery phase of the DSS-induced IBD observed with HE staining and Alcian blue staining, respectively. **(J1,K1)** The normal group: no significant change was observed in the colonic mucosa; **(J2,K2)** the DSS group: ulcer of the colonic mucosa was replaced by an inflammatory scar; **(J3,K3)** the DSS + *B. subtilis*-fermented milk group: the epithelium was almost integrated and the surficial ulcer was replaced by regenerated regenerative colonic glands consisting of goblet cells, which were filled with abundant mucus. **(L)** Histological damage scores of different groups evaluated with H&E-stained sections of the small intestines. The histological damage scores of the DSS + *B. subtilis*-fermented milk group in both the active phase and the recovery phase were significantly lower than those of the DSS group (*n* = 5, * represents *p* < 0.05). **(M)** Histological damage scores of different groups evaluated with H&E-stained sections of the colons. The histological damage scores of the DSS + *B. subtilis*-fermented milk group in both the active phase and the recovery phase were significantly lower than those of the DSS group (*n* = 5, ** represents *p* < 0.01).

For evaluation of the disease severity of DSS-induced IBD, the DAI scoring was performed and the length of the colon was measured. As shown in Figure 1A, during the second 7-day period (the active phase of IBD), the DAI of the DSS group, the DSS + milk group, and the DSS + *B. subtilis*-fermented milk group increased continuously as the DSS intake was extended. However, the DAI in the DSS + *B. subtilis*-fermented milk group were lower than those of the DSS group and the DSS + milk group. In the third 7-day period (the recovery phase of IBD), after termination of DSS intake, the recovery of DAI with varying degrees was observed in the DSS group, the DSS + milk group, and the DSS + *B. subtilis*-fermented milk group. However, in the DSS + *B. subtilis*-fermented milk group, the DAI index recovered faster and was significantly lower than those of the DSS group and the DSS + milk group. It was suggested that the *B. subtilis*-fermented milk could effectively lessen the symptoms of the DSS-induced IBD in the active phase. Moreover, after termination of DSS irritation, recovery from the DSS-induced injury was also promoted by oral intake of the *B. subtilis*-fermented milk. These results suggested that oral intake of DSS could induce injury of the intestines; however, oral administration of the *B. subtilis-*fermented milk could significantly lessen the symptoms of DSS-induced IBD. As shown in Figures 1B,C, at 7 days after the termination of DSS intake, the colons in the DSS group and the DSS + milk group were shortened significantly compared with those in the normal group and *B. subtilis*-fermented milk group. However, in the DSS + *B. subtilis*-fermented milk, the colons were shortened only slightly and were significantly longer than those of the DSS group and the DSS + milk group. It was suggested that the *B. subtilis*-fermented milk could reduce the injury of the colons induced by DSS. In this experiment, the *B. subtilis*-fermented milk group showed no obvious difference from the normal group, and the DSS + milk group showed no obvious difference from the DSS group, so that the *B. subtilis*-fermented milk group and the DSS + milk group were dismissed in the following sections about pathological observation and cell cytokine detection.

### Effect of the *B. subtilis*-Fermented Milk on the Pathological Changes in the Small Intestine and the Colon

The tissue sections of the small intestines and the colons in the different groups were stained with eosin–hematoxylin and Alcian blue. The pathological changes of the intestinal mucosa in the active phase and recovery phase of the IBD were observed and compared.

#### Effect of the *B. subtilis*-Fermented Milk on the Pathological Changes in the Small Intestine

In the small intestine, the histological damage score (Figure 1L) of the DSS group was 5.2 ± 0.8 in the active phase, which significantly reduced to 2.9 ± 0.6 in the recovery phase. The histological damage score of the DSS + *B. subtilis*-fermented milk group was 3.3 ± 0.5 in the active phase, which significantly reduced to 1.4 ± 0.6 in the recovery phase. The scores of the DSS + *B. subtilis*-fermented milk group in both phases were significantly lower than those of the DSS group.

As shown in Figures 1D1,E1, for normal mice, the small intestinal mucosa was integrated, with the intestinal villi and the crypts arranged well. The epithelial cells covering the villi were closely arranged with no defects. The goblet cells with abundant mucus (blue) were observed in the villi and crypts. For the DSS group, at 7 days after oral intake of DSS (the active phase of IBD), villi disintegration, epithelial exfoliation, and glandular structure destruction were observed in the small intestinal mucosa due to formation of inflammatory ulcer induced by DSS (Figures 1D2,E2); at 7 days after the termination of DSS intake (the recovery phase of IBD), the damage in the small intestinal mucosa recovered partially, but the villus structure was still disordered, and the epithelium was not intact (Figures 1F2,G2). However, for the DSS + *B. subtilis*-fermented milk group, after 7 days of DSS intake (the active phase of IBD, Figures 1D3,E3), the damage of the small intestinal mucosa was slight. Local superficial ulcer and partial abscission of the epithelial cells were observed. The crypt structure was almost intact and abundant mucus was observed in the goblet cells; at 7 days after termination of DSS intake (the recovery phase of IBD, Figures 1F3,G3), the ulcer was repaired by regenerated epithelium and the intact epithelial barrier covering the mucosa was already reconstructed. The regenerative villus arrangement was still relatively scattered compared with the villus in the normal small intestine, but the recovery of the intestinal epithelium in the DSS + *B. subtilis*-fermented milk was obviously better than that of the DSS group. These results suggested that oral intake of *B. subtilis*-fermented milk could not only prevent the DSS-induced damage of the small intestinal mucosa in the active phase of the IBD, but also promote repairing of the injury in the recovery phase of IBD.

#### Effect of the *B. subtilis*-Fermented Milk on the Pathological Changes in the Colon

In the colon, the histological damage scores (Figure 1M) of the DSS group were 7.1 ± 0.7 in the active phase and 6.6 ± 0.9 in the recovery phase. The histological damage scores of the DSS + *B. subtilis*-fermented milk group were 3.7 ± 0.7 in the active phase and 2.3 ± 0.6 in the recovery phase, which were significantly lower than those of the DSS group.

As shown by the histological sections, in the normal colon (Figures 1H1,I1), the epithelial cells were closely arranged with no local defects. The goblet cells with abundant mucus (blue) were observed in the glands. For the DSS group, after 7 days of administration of DSS (Figures 1H2,I2), the damage of the colonic mucosa was more severe than that of the small intestine shown in Figures 1D2,E2. Bleeding and deep ulcer were observed obviously. The epithelial cells and goblet cells were mostly destroyed. There was only scattered distribution of crypt epithelial cells and goblet cells in the ulcer. At 7 days after termination of DSS intake (Figures 1J2,K2), the local epithelium was lost completely, and the scar was formed due to hyperplasia of the inflammatory tissue. The scar tissue was exposed to the lumen without epithelial cell covering. However, for the DSS + *B. subtilis*-fermented milk group, the damage in the colonic mucosa was slighter than that of the DSS group. The surviving glands and goblet cells were observed (Figure 1H3), and there was abundant mucus in the goblet cells (Figure 1I3); at 7 days after termination of DSS intake, the damage in the colonic mucosa was obviously recovered, and there were regenerative epithelial cells and glands in the colonic mucosa (Figures 1J3,K3). These results suggested that oral administration of the *B. subtilis*-fermented milk could significantly reduce the DSS-induced damage of the colonic mucosa in the active phase of the IBD, and promote the regeneration of the epithelial cells and glands of the injured colonic mucosa in the recovery phase of IBD.

### Effect of the *B. subtilis*-Fermented Milk on the Neutrophil Infiltration and Expression of the Inflammatory/Anti-inflammatory Cytokines

**FIGURE 2:**
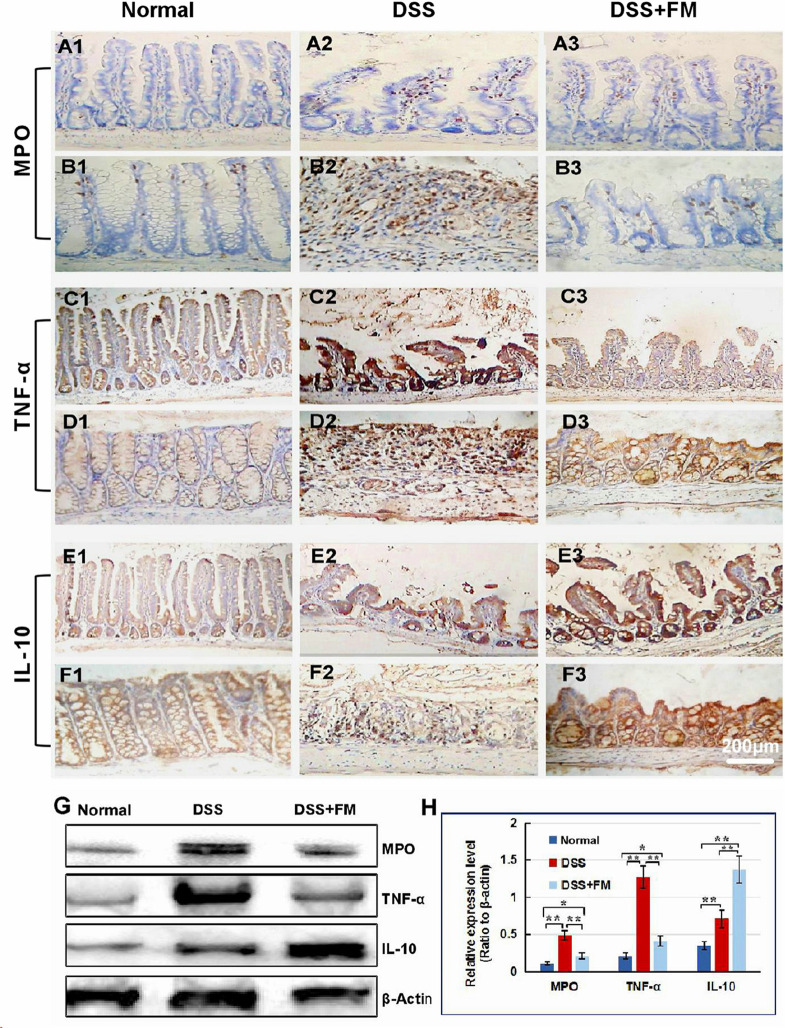
The infiltration of MPO^+^ neutrophils, and the cellular distribution and relative expression level detection of the TNF and IL-10 in the small intestinal and colonic mucosa at 7 days after the termination of DSS administration. **(A)** The MPO immunohistochemistry staining of the small intestinal mucosa: **(A1)** the normal group: few neutrophils were observed in the small intestinal mucosa; **(A2)** the DSS group: a number of accumulative MPO^+^ neutrophils (brown) infiltrated into the mucosa epithelium; **(A3)** the DSS + *B. subtilis-*fermented milk group: only limited neutrophil infiltration could be observed in the small intestinal mucosa. **(B)** The MPO immunohistochemistry staining of the colonic mucosa: **(B1)** the normal group: few neutrophils were observed in the colonic mucosa; **(B2)** the DSS group: colonic epithelium and the glands disappeared, and the ulcer was locally replaced by scars and a number of accumulative MPO^+^ neutrophils (brown) were observed in the scars; **(B3)** the DSS + *B. subtilis*-fermented milk group: only limited MPO^+^ neutrophils observed in the colonic mucosa. **(C)** The TNF immunohistochemistry staining of the small intestinal mucosa: **(C1)** the normal group: the epithelium was integrated with faint yellow staining, suggesting low expression of TNF; **(C2)** the DSS group: the villus structure is not integrated, and the epithelial cells showed black brown, suggesting overexpression of TNF; **(C3)** the DSS + *B. subtilis*-fermented milk group: the villus and the glands were almost integrated, and the staining of epithelial cells was similar to that of the normal group **(C1)**, suggesting low expression of TNF. **(D)** The TNF immunohistochemistry staining of the colonic mucosa: **(D1)** the normal colonic mucosa: the epithelium was integrated with low TNF expression (faint yellow); (**D2**) the DSS group: the epithelium structure and the glands were destroyed and replaced by a scar, and there were a number of TNF^+^ inflammatory cells (black brown) in the scar; **(D3)** the DSS + *B. subtilis*-fermented milk group: the recovered epithelium showed faint yellow, suggesting low TNF expression. **(E)** The IL-10 immunohistochemistry staining of the small intestinal mucosa: **(E1)** the normal small intestinal mucosa: the IL-10 staining dispersed in the villi and the crypts with faint yellow, suggesting low-level expression of IL-10; **(E2)** the DSS group, the residual epithelium and the crypts were light brown, suggesting mid-level of IL-10 expression; **(E3)** the DSS + *B. subtilis*-fermented milk group: the dark brown staining of the regenerative epithelium represented high-level expression of IL-10. **(F)** The IL-10 immunohistochemistry staining of the colonic mucosa: **(F1)** the normal group: the IL-10 staining dispersed in the glands with bright yellow, suggesting low-level expression of IL-10; **(F2)** the DSS group: there were few IL-10^+^ cells in the scars; **(F3)** the DSS + *B. subtilis*-fermented milk group, the dark brown staining of the epithelial cells represented high-level expression of IL-10. **(G,H)** Western blotting analysis for the expression of MPO, TNF, and IL-10 in the samples containing equivalent ileum and colon. The expression level of MPO, TNF, and IL-10 in the DSS group was significantly higher than that of the normal (control) group. The expression level of MPO and TNF in the DSS + *B. subtilis*-fermented milk (FM) group was significantly lower than that of the DSS group, while the expression level of IL-10 in the DSS + *B. subtilis*-fermented milk (FM) group was significantly higher than that of the DSS group (*n* = 5, * represents *p* < 0.05, ** represents *p* < 0.01).

For the evaluation of inflammatory neutrophil infiltration in the intestinal mucosa, immunohistochemistry staining of neutrophil-specific MPO was used to show the neutrophils. As shown in Figure 2A1, in the normal small intestinal mucosa, few MPO^+^ neutrophils were observed. For the DSS group (Figure 2A2), massive accumulative MPO^+^ neutrophils were observed beneath the mucosa epithelium at 7 days after termination of intake of DSS; for the DSS + *B. subtilis*-fermented milk group (Figure 2A3), however, only limited neutrophils could be observed in the small intestinal mucosa at 7 days after termination of intake of DSS. Compared with the small intestine (Figure 2A), a similar difference, between the DSS group and the DSS + *B. subtilis*-fermented milk group, was observed in the colonic mucosa (Figure 2B). In the normal colonic mucosa (Figure 2B1), few neutrophils were observed. For the DSS group (Figure 2B2), at 7 days after the termination of DSS administration, the colonic epithelium and the glands disappeared, and the ulcer was locally replaced by scars and massive accumulative MPO^+^ neutrophils were observed in the scars. However, in the DSS + *B. subtilis*-fermented milk group (Figure 2B3), only limited neutrophils could be observed in the regenerative colonic mucosa. These results suggested that oral administration of the *B. subtilis*-fermented milk could significantly reduce inflammatory severity of DSS-induced IBD. To explore the anti-inflammatory mechanisms of the *B. subtilis*-fermented milk, the pro-inflammatory cytokine TNF and anti-inflammatory cytokine IL-10 were stained with IHC. In normal small intestinal mucosa (Figure 2C1), the villus epithelium was integrate with low TNF expression. For the DSS group (Figure 2C2), at 7 days after the termination of DSS administration, the villus structure is not integrated, with high TNF expression. For the DSS + *B. subtilis*-fermented milk group (Figure 2C3), the villus and the glands were almost integrated, and the relative expression level of TNF was significantly lower than that of the DSS group. It was suggested that administration of the *B. subtilis*-fermented milk could significantly reduce TNF expression in the small intestine. Compared with the small intestine (Figure 2C), a similar difference, between the DSS group and the DSS + *B. subtilis*-fermented milk group, was also observed in the colon (Figure 2D). For the normal colonic mucosa (Figure 2D1), the epithelium and the colonic glands were integrated with low TNF expression; for the DSS group (Figure 2D2), at 7 days after the termination of DSS administration, the epithelium structure and the glands were destroyed and replaced by the inflammatory scars, which overexpressed TNF. For the DSS + *B. subtilis*-fermented milk group (Figure 2D3), the colonic epithelium and the glands were almost integrated, and the relative expression level of TNF was significantly lower than that of the DSS group. As shown in Figures 2E,F, in the normal small intestinal mucosa (Figure 2E1) and colonic mucosa (Figure 2F1), the middle immunohistochemistry staining of IL-10 was mainly located in the small intestinal crypts and colonic glands. For the DSS group (Figures 2E2,F2), IL-10 staining was observed in the residual small intestinal epithelium and the crypts (Figure 2E2). In the inflammatory scar of colonic mucosa, there were a few inflammatory cells with positive IL-10 staining (Figure 2F2); for the DSS + *B. subtilis*-fermented milk group (Figures 2E3,F3), the dark brown staining in the regenerative epithelium of the small intestinal mucosa (Figure 2E3) and colonic mucosa (Figure 2F3) represented a high-level expression of IL-10. For quantification analysis of the expressions of MPO, TNF, and IL-10, western blotting was used to show the relative expression levels of these proteins. Figures 2G,H show that the relative expression level of MPO, TNF, and IL-10 in the DSS group was significantly higher than that of the normal group. The expression of MPO and TNF in the DSS + *B. subtilis*-fermented milk group was significantly lower than that of the DSS group, while the expression of IL-10 in the DSS + *B. subtilis*-fermented milk group was significantly higher than that of the DSS group. These results suggested that the *B. subtilis*-fermented milk could inhibit the expression of pro-inflammatory cytokine TNF and promote the expression of anti-inflammatory cytokine IL-10 in the mucosa of the small intestine and colon of the DSS-induced IBD animal models.

### Effect of the *B. subtilis*-Fermented Milk on the Expression of Lgr5, CDX2, and Mucin2

**FIGURE 3:**
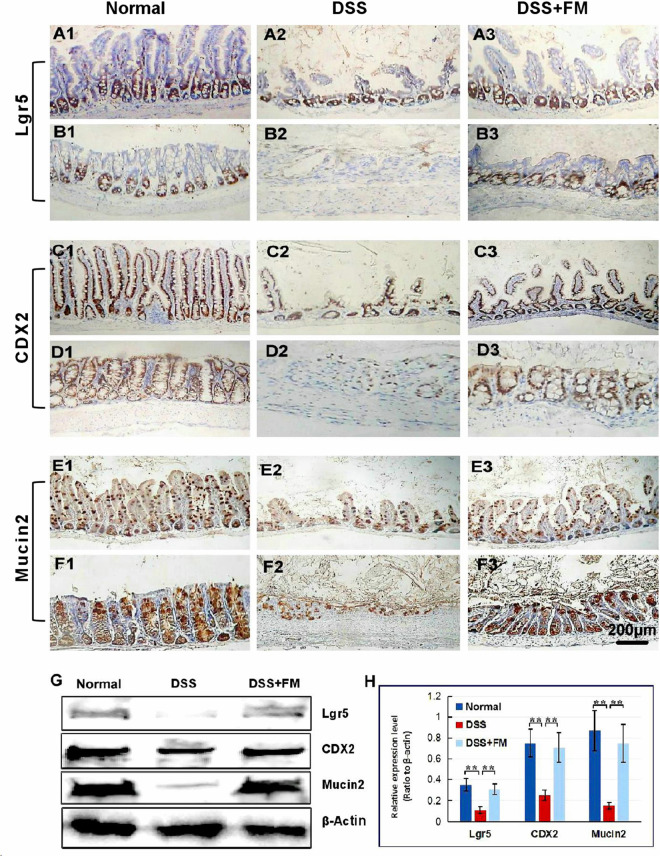
The distribution of Lgr5^+^ ISCs in the intestinalmucosa and the subcellular localization and relative expression level detection of epithelial function proteins CDX2 and villin in the intestinal mucosa of IBD at 7 days after termination of DSS administration. **(A)** The Lgr5^+^ ISCs (brown) in the small intestinal mucosa: **(A1)** the normal group, the villi and the crypts were arranged compactly, and Lgr5^+^ ISCs were observed in the crypts; **(A2)** the DSS group, the villi and the crypts were scattered, with few Lgr5^+^ ISCs; **(A3)** the DSS + *B. subtilis*-fermented milk group, there were more Lgr5^+^ ISCs in villi and crypts compared with those in the DSS group. **(B)** The Lgr5^+^ ISCs (brown) in the colonic mucosa: **(B1)** the normal group, the glands were arranged compactly, and there were large amounts of Lgr5^+^ ISCs at the bottom of the glands; **(B2)** the DSS group: the ulcers were replaced by scars. No Lgr5^+^ ISCs were observed in the scars; **(B3)** the DSS + *B. subtilis*-fermented milk group: the colonic epithelium was integrated, with some regenerated glands. A number of Lgr5^+^ ISCs were observed at the bottom of the regenerated glands. **(C)** The CDX2 was localized in the epithelial cellular nuclei (brown) by immunohistochemistry staining in the small intestinal mucosa: **(C1)** the normal group: the villi and the crypts were arranged compactly, and CDX2^+^ epithelial cells were observed on the surface of the villi and the crypts; **(C2)** the DSS group: the villi and the crypts were scattered, and few CDX2^+^ epithelial cells were observed on the surface of the crypt and the villi; **(C3)** the DSS + *B. subtilis*-fermented milk group: more villi and crypts were observed in comparison with the DSS group, and there were more CDX2^+^ epithelial cells covering the villi and crypts. **(D)** The CDX2 was localized in the epithelial cellular nuclei (brown) by immunohistochemistry staining in the colonic mucosa: **(D1)** the normal group: the colonic glands were arranged compactly, and CDX2^+^ epithelial cells were observed on the surface of the glands; **(D2)** the DSS group: the glands were scattered, and few CDX2^+^ epithelial cells were observed in the scar; **(D3)** the DSS + *B. subtilis*-fermented milk group: more colonic glands were observed in comparison with the DSS group, and there were more CDX2^+^ epithelial cells in the glands. **(E)** The Mucin2 was localized in the cytoplasm of the goblet cells (brown) by immunohistochemistry staining in the small intestinal mucosa: **(E1)** the normal group, a number of Mucin2^+^ goblet cells observed in the epithelium; **(E2)** the DSS group: only few Mucin2^+^ goblet cells were observed in the remaining villi and crypts; **(E3)** the DSS + *B. subtilis*-fermented milk group: more Mucin2^+^ goblet cells were observed in the recovered mucosa. **(F)** The Mucin2 was localized in the cytoplasm of the goblet cells (brown) by immunohistochemistry staining in the colonic mucosa: **(F1)** the normal group, large amounts of Mucin2^+^ goblet cells were observed in the mucosa; **(F2)** the DSS group: only few Mucin2^+^ goblet cells were observed in the scars; **(F3)** the DSS + *B. subtilis*-fermented milk group: more Mucin2^+^ goblet cells were observed in the recovered colonic mucosa. **(G,H)** Western blotting was applied for detection of the relative expression level of Lgr5, CDX2, and Mucin2 in the samples containing equivalent ileum and colon. The expression level of Lgr5, CDX2, and Mucin2 in the DSS group was significantly lower than that of the normal (control) group. The expression level of Lgr5, CDX2, and Mucin2 in the DSS + *B. subtilis*-fermented milk (FM) group was significantly higher than that of the DSS group (*n* = 5, ** represents *p* < 0.01).

The Lgr5 is the marker of the intestinal stem cells, which could differentiate to epithelial cells and goblet cells of the mucosa in the small intestine and the colon (Umar, 2010). In the normal small intestinal mucosa, Lgr5^+^ stem cells were observed in the crypts (Figure 3A1). For the DSS group, at 7 days after termination of DSS intake, Lgr5^+^ stem cells reduced significantly and were observed in the bottom of the crypts in the small intestinal mucosa (Figure 3A2). For the DSS + *B. subtilis*-fermented milk group, at 7 days after termination of DSS intake, more Lgr5^+^ stem cells were observed in the crypts of the small intestinal mucosa (Figure 3A3). Meanwhile, in the normal colonic mucosa, Lgr5^+^ stem cells were located deep in the colonic glands (Figure 3B1). For the DSS group, at 7 days after termination of DSS intake, the Lgr5^+^ stem cells almost disappeared due to the formation of inflammatory ulcer and scar in the colonic mucosa (Figure 3B2). For the DSS + *B. subtilis*-fermented milk group, at 7 days after termination of DSS intake, a number of Lgr5^+^ stem cells were observed in the colonic mucosa (Figure 3B3). For evaluation of the ability of the Lgr5^+^ stem cells to differentiate into epithelial cells and goblet cells, the epithelium cell marker CDX2 and the goblet cell marker Mucin2 were stained with IHC (Deplancke and Gaskins, 2001; Sun et al., 2017). In the normal small intestinal mucosa, CDX2^+^ epithelial cells covered the villi and crypts and formed an integrated epithelial barrier (Figure 3C1). For the DSS group, the villi and the glands were scattered due to the inflammatory damage, and few CDX2^+^ epithelial cells were observed in the residual villi and crypts (Figure 3C2). For the DSS + *B. subtilis*-fermented milk group (Figure 3C3), more regenerative villi and crypts were observed and there were more CDX2^+^ epithelial cells covering the villi and crypts in comparison with the DSS group. Meanwhile, in the normal colonic mucosa (Figure 3D1), the CDX2^+^ epithelial cells were located densely in the colonic glands. For the DSS group (Figure 3D2), at 7 days after termination of DSS intake, there were a few CDX2^+^ epithelial cells in the inflammatory scar of the colonic mucosa. For the DSS + *B. subtilis*-fermented milk group (Figure 3D3), at 7 days after termination of DSS intake, a number of CDX2^+^ epithelial cells were observed in the regenerative glands of the colonic mucosa. Mucin2 is a major intestinal *O*-glycosylated protein secreted by goblet cells. This secreted protein formed an important physiological barrier for host defense against pathogenic bacteria. For the normal group, large amounts of Mucin2^+^ goblet cells were observed in the small intestinal mucosa (Figure 3E1) and the colonic mucosa (Figure 3F1). For the DSS group, only few Mucin2^+^ goblet cells were observed in the remaining villi in the small intestine (Figure 3E2) and the scars in the colon (Figure 3F2). For the DSS + *B. subtilis*-fermented milk group, large amounts of Mucin2^+^ goblet cells were observed in the regenerative mucosa in both the small intestine (Figure 3E3) and the colon (Figure 3F3). These results suggested that the integrative epithelial and mucous barrier covering the intestinal mucosa were recovered due to intake of the *B. subtilis*-fermented milk. In accordance with the above results, the western blotting results (Figures 3G,H) showed that the relative expression levels of Lgr5, CDX2, and Mucin2 in the DSS group were significantly lower than those of the normal group. The relative expression levels of these proteins in the DSS + *B. subtilis*-fermented milk group were significantly higher than those of the DSS group. These results indicated that the *B. subtilis*-fermented milk could induce the expression of these proteins, resulting in the regeneration of the epithelium lining the intestinal mucosa injured by DSS-induced IBD.

### Effect of the *B. subtilis*-Fermented Milk on the Expression of ZO-1 and Villin

**FIGURE 4:**
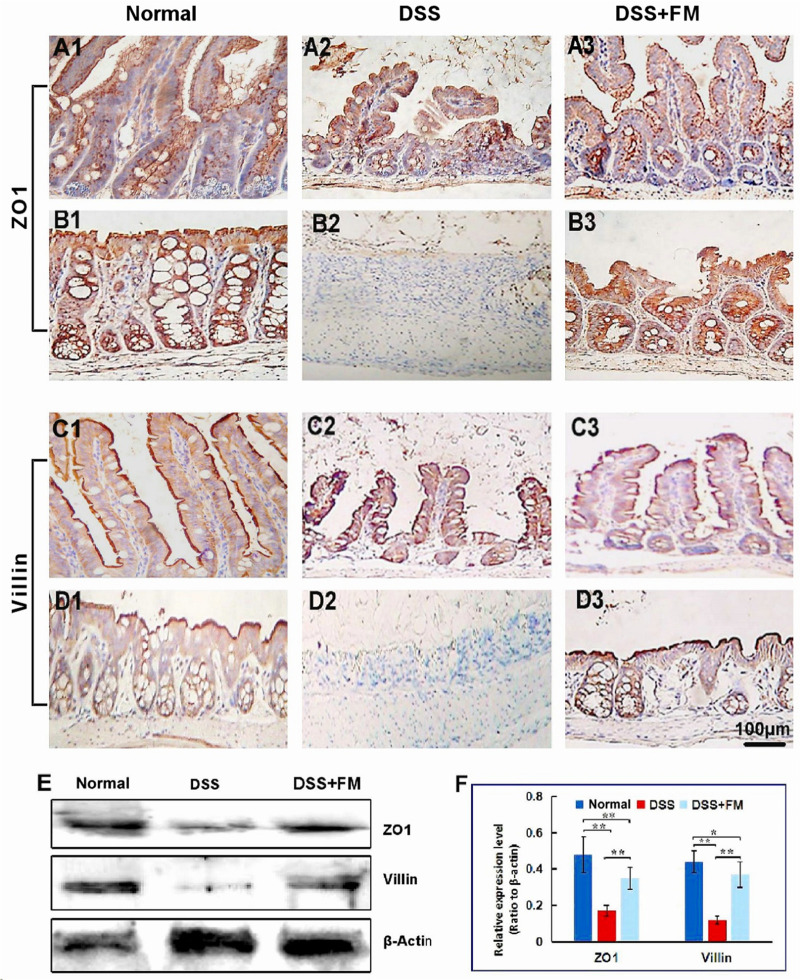
The subcellular localization and relative expression level detection of ZO-1 and villin in the intestinal mucosa of IBD at 7 days after termination of DSS intake. **(A)** The ZO-1 immunohistochemistry staining of the small intestinal epithelial TJP (brown dots): **(A1)** the normal group: the villi and crypts were arranged compactly, and the ZO-1-positive staining (representing the TJP) showed the dotted line (brown) along the surface of the villi and the crypts; **(A2)** the DSS group: ZO-1 distributed dispersively in the residual villi of the small intestinal mucosa; **(A3)** the DSS + *B. subtilis*-fermented milk group: the ZO-1 staining formed the dotted line (brown, representing the TJP) at the subsurface of the regenerative villi. **(B)** The ZO-1 immunohistochemistry staining of the colonic epithelial TJP (brown dots): **(B1)** the normal group: ZO-1-positive staining distributed on the inner side of the epithelial cell membrane (representing the TJP); **(B2)** the DSS group: there was no ZO-1-positive staining in the scar; **(B3)** the DSS + *B. subtilis*-fermented milk group: the ZO-1-positive staining distributed on the inner side of the regenerative epithelial cell membrane (representing the TJP). **(C)** The villin immunohistochemistry staining (brown strip) of the small intestinal microvilli: **(C1)** the normal group: villin-positive staining showed a strip-like distribution on the surface of the villi in the normal small intestinal mucosa; **(C2)** the DSS group: villin distributed at the surface of the residual villi; **(C3)** the DSS + *B. subtilis*-fermented milk group: villin-positive staining formed an integrative strip (brown) enclosing the surface of the regenerative villi. **(D)** The villin immunohistochemistry staining of the colonic epithelium: **(D1)** the normal group: villin-positive staining (brown) showed banded distribution on the surface of the epithelium; **(D2)** the DSS group: almost no villin-positive staining was observed in the scar due to damage of the epithelium; **(D3)** the DSS + *B. subtilis*-fermented milk group: the villin-positive staining (brown) showed banded distribution on the surface of the regenerated epithelium in the colonic mucosa. **(E,F)** The western blotting analysis for the relative expression level of ZO-1 and villin in the samples contained equivalent ileum and colon. The expression level of ZO-1 and villin in the DSS group was significantly lower than that of the normal (control) group. The expression level of ZO-1 and villin and in the DSS + *B. subtilis*-fermented milk (FM) group was significantly higher than that of the DSS group (*n* = 5, * represents *p* < 0.05, ** represents *p* < 0.01).

ZO-1 is the marker protein of tight junction of the intestinal epithelium, and villin is the marker protein of the microvilli located in the free surface of the epithelial cells. These proteins play an important role in maintaining the integrity of the epithelium barrier (Hodin et al., 1997; Tan et al., 2019). The image of IHC staining for ZO-1 in Figure 4A1 showed that, in the normal small intestinal mucosa, the villi and the crypts were arranged compactly, and the ZO-1 showed a dotted-line-like distribution (the dots represented tight junction between the epithelial cells) along the surface of the villi and the crypts. For the DSS group (Figure 4A2), at 7 days after termination of DSS intake, ZO-1 distributed dispersively in the residual epithelium of the small intestinal mucosa. However, in the DSS + *B. subtilis-*fermented milk group (Figure 4A3), the ZO-1 distribution was similar to that of the normal small intestine and showed a dotted-line-like distribution along the surface of the newborn villi, which suggested that the new tight junctions between the regenerative epithelial cells were reconstructed. Meanwhile, in the normal colonic mucosa (Figure 4B1), the ZO-1 was located mainly at the membrane of the epithelial cells. For the DSS group (Figure 4B2), there was no obvious ZO-1^+^ cell in the colonic mucosa due to the formation of inflammatory ulcer and scar in the colonic mucosa. However, for the DSS + *B. subtilis*-fermented milk group (Figure 4B3), the ZO-1 was located mainly at the membrane of the regenerative epithelial cells of the colonic mucosa. For IHC staining of the villin, which showed a strip-like distribution on the surface of the normal small intestinal mucosa due to its distribution in the microvilli of the epithelial cells, the strip represented the striated border consisting of the microvilli (Figure 4C1). In the DSS group, villin was distributed on the surface of the residual villi of the small intestinal mucosa (Figure 4C2). However, in the DSS + *B. subtilis*-fermented milk group, villin-positive staining formed an integrative strip enclosing the surface of the regenerative villi of the small intestinal mucosa (Figure 4C3). Meanwhile, in the normal colonic mucosa, villin was distributed at the surface of the epithelium (Figure 4D1). In the DSS group, there were no villin^+^ cells in the scar of the colonic mucosa (Figure 4D2). In the DSS + *B. subtilis*-fermented milk group, villin-positive microvilli showed a strip-like distribution on the surface of the colonic epithelium (Figure 4D3). According to the western blotting results (Figures 4E,F), the relative expression levels of ZO-1 and villin in the DSS group were significantly lower than those of the normal group. The relative expression levels of ZO-1 and villin in the DSS + *B. subtilis*-fermented milk group were significantly higher than those of the DSS group. These results suggested that the oral intake of the *B. subtilis*-fermented milk could induce the small intestinal and colonic epithelium to overexpress the ZO-1 and villin, which promoted the formation of a tight junction between the intestinal epithelial cells and maintained the integrity of the epithelium barrier.

### Effect of the *B. subtilis*-Fermented Milk on the Intestinal Flora of DSS-Induced IBD Animal Models

**FIGURE 5:**
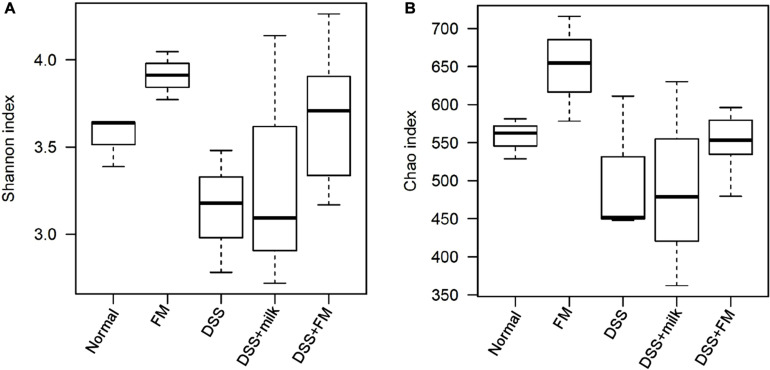
The α-diversity of the intestinal flora indicated by the Shannon index and the Chao index. **(A)** Shannon index of the OTU detected in different groups (*n* = 5). **(B)** Chao index of the OTU detected in different groups (*n* = 5).

The etiology of IBD remains unknown. However, one of the main causes is likely related to the imbalance in the gut microbiota. Changes in the microbiota have been observed in previous studies, and decreased bacterial diversity and increased bacterial instability were verified in patients with IBD compared with healthy individuals. In this study, DSS definitely disrupted the balance of the intestinal flora and altered the diversity and composition of the gut microbiota. The α-diversity Shannon index and Chao index (Figure 5) showed that the flora diversity of the *B. subtilis*-fermented milk group was significantly higher than that of the normal group, which suggested that the introduction of *B. subtilis* enhanced the diversity and the balance of the intestinal flora. The Shannon index and the Chao index of the intestinal microbiota were significantly lower in the DSS group than those of the normal group. It was suggested that DSS-induced IBD is related with enteric dysbacteriosis. The average values of the Shannon index and the Chao index of the DSS + milk group were similar to those of the DSS group and were lower than those of the *B. subtilis*-fermented milk group, although the differences of both indexes were not statistically significant due to the large deviations. It was suggested that the oral intake of milk did not show a significant rise of the Shannon index and the Chao index. The Shannon index and the Chao index of the DSS + *B. subtilis*-fermented milk group were significantly higher than those of the DSS group, which suggested that intake of *B. subtilis* could maintain the intestinal microbiota diversity.

**FIGURE 6:**
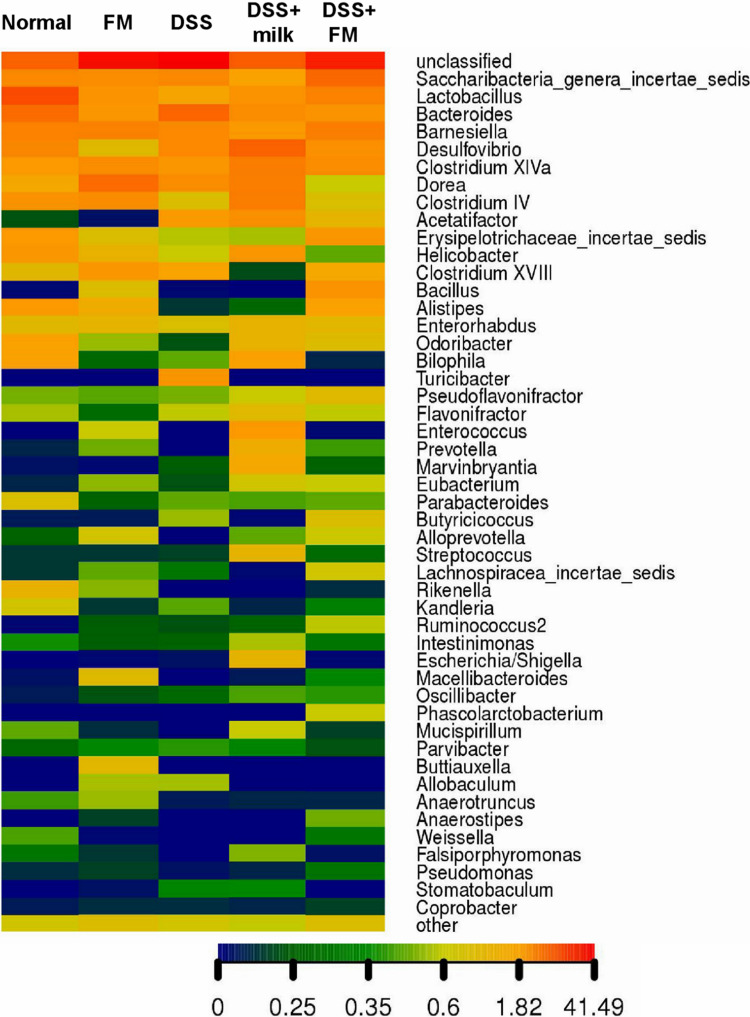
The genus heatmap of the intestinal flora at 7 days after termination of DSS intake. Group 1, normal group; group 2, *B. subtilis*-fermented milk group; group 3, DSS group; group 4, DSS + milk group; group 5, DSS + *B. subtilis*-fermented milk group (*n* = 5). Different colors represent different percentages of the individual genus in the total OTU.

The abundance heatmap of the flora on the genus level is shown in Figure 6. In the *B. subtilis*-fermented milk group, the abundance of *Bacillus*, *Enterococcus*, *Alloprevotella*, *Ruminococcus*, and *Buttiauxella* was significantly higher than that in the normal group. In the DSS group, the abundance of multiple bacteria (including *Alistipes*, *Rikenella*, *Barnesiella*, *Macellibacteroides*, and *Lactobacillus*) was significantly decreased in comparison with the normal group, while the abundance of *Escherichia* and *Bacteroides* increased dramatically. The flora composition of the DSS + *B. subtilis*-fermented milk group was very different from that of the DSS group. The abundance of *Bacillus*, *Alloprevotella*, and *Ruminococcus* was higher than those of the normal group and the DSS group. The abundance of *Alistipes* and *Lactobacillus* in the DSS + *B. subtilis*-fermented milk group was much higher than that in the DSS group, but slightly lower than that in the normal group.

**FIGURE 7:**
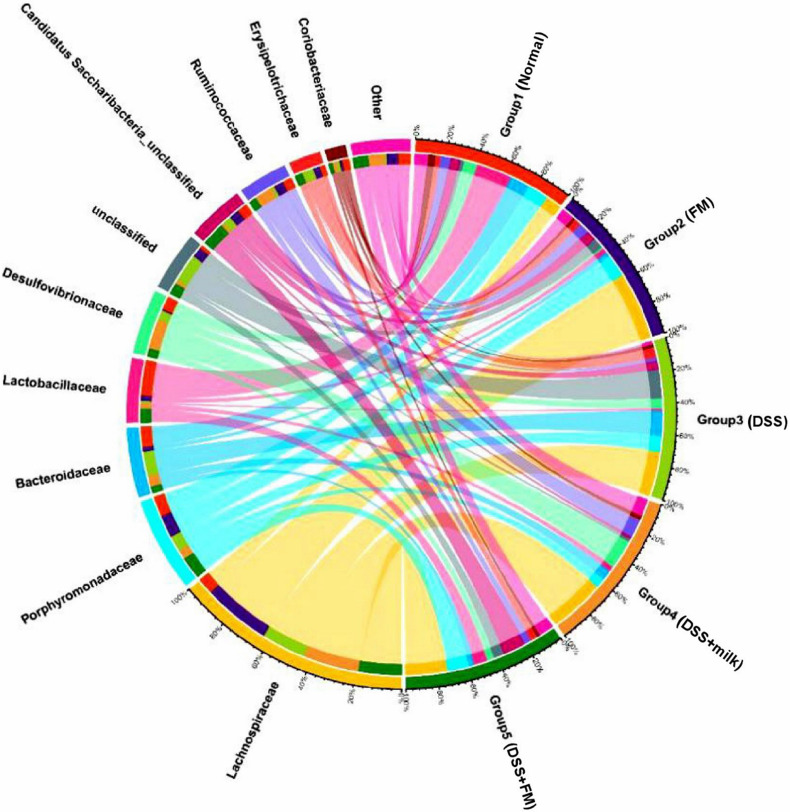
Microbial distribution at the family level. Group 1, normal group; group 2, *B. subtilis*-fermented milk group; group 3, DSS group; group 4, DSS + milk group; group 5, DSS + *B. subtilis*-fermented milk group (*n* = 5).

The dominant bacteria on a family level is shown in Figure 7, and the dominant bacteria in the normal group included *Lactobacillae*, *Porphyromonadaceae*, *Bacteroidaceae*, *Lachnospiraceae*, and *Desulfovibrionaceae*. In the DSS group, the dominant families were *Lachnospiraceae* and *Bacteroidaceae*. Compared with the DSS group, the fractions of *Lachnospiraceae* and *Bacteroidaceae* were significantly reduced in the DSS + *B. subtilis*-fermented milk group, and the fraction of *Lactobacillae* was largely increased.

**FIGURE 8:**
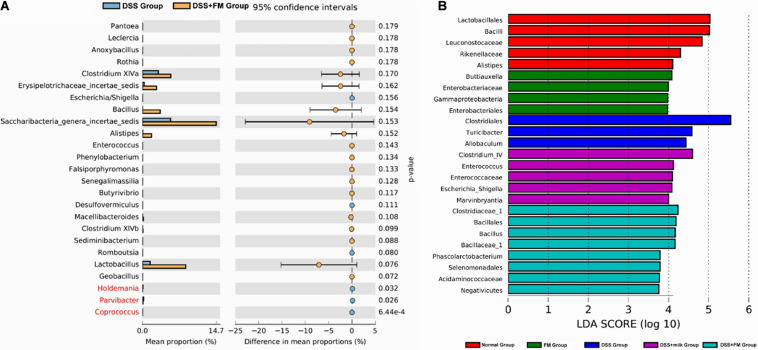
Difference in the mean proportions of the major composition of the intestinal flora. **(A)** Mean proportions of the top 25 genus in the intestinal flora at 7 days after termination of DSS intake and the statistical difference between group 3 (DSS group) and group 5 (DSS + *B. subtilis*-fermented milk group) (*n* = 5). **(B)** Significantly different taxa as measured by LEfSe analysis (threshold > 3.5).

The difference analysis in mean proportions (Figure 8A) showed that the abundance of *Bacillus*, *Barnesiella*, *Alistipes*, and *Saccharibacteria* was higher in the DSS + milk group than that in the DSS group. According to the LEfSe algorithm analysis (Figure 8B), the abundance of *Clostridiales*, *Turicibacter*, and *Allobaculum* was significantly higher in the DSS group than that in the normal group. For the DSS + *B. subtilis*-fermented milk group, the abundance of *Clostridiaceae_1*, *Bacillus*, *Bacillales*, *Bacillaceae_1*, *Phascolarctobacterium*, and *Selenomonadales* was significantly higher than those of the other groups. The abnormal high fractions of *Clostridales*, *Turicibacter*, and *Allobaculum* in the DSS group were regulated in the DSS + *B. subtilis*-fermented milk group. These results suggested that oral intake of *B. subtilis*-fermented milk could efficiently increase the flora diversity of the intestinal microbiota and restore the balance of gut flora which was disturbed by DSS.

## Discussion

Dextran sulfate sodium salt is widely used to construct the IBD animal model. It has been assumed in most former researches that DSS induces epithelial damage mainly in the colon and pathological change in the small intestine is ignored (Elsheikh et al., 2012; Kim et al., 2012). In this research, however, it was observed that DSS induced damage in both the small intestine and the colon when it was given orally in drinking water. Local mucosa of the small intestine and the colon was damaged by ulcer and hemorrhagic necrosis, along with inflammatory cell infiltration. This result was in agreement with the report from Elsheikh et al. (2012) and suggested that the DSS-induced IBD was more similar to CD in pathological features. In comparison with the colon, the small intestine is longer, with larger mucosa surface, which is favorable for nutrition adsorption. The damage of the small intestine mucosa would lead to innutrition, which could cause various morbidities including weight loss and the decrease of immunological defense (Peuhkuri et al., 2010). Thus, the protection of the small intestinal structure and function is more important than protection of the colon. Based on the pathological characteristics of IBD, the prevention and curation of IBD should focus on the inhibition of inflammatory reaction and the protection of the mucosa both in the small intestine and in the colon from inflammatory injury. Because the CD, especially small-bowel Crohn’s disease, possessed higher disease severity and more extensive injury in the intestinal mucosa (including the small intestine and colon) than UC (Cahill et al., 2014; Fong and Irving, 2015; Al Nabhani et al., 2020; Du et al., 2020), protection of the small intestine from inflammatory damage and promotion of mucosal healing in the small intestine of IBD are more effective for the prevention and curation of this disease especially for small-bowel Crohn’s disease. Meanwhile, the promotion of epithelial stem cell proliferation and differentiation and the reconstruction of the integrated mucosa barrier are essential for recovery from IBD. Besides the traditional therapy of anti-inflammatory drugs, the application of regulators for the gut flora is a novel therapeutic strategy in preventing recurrence and ameliorating refractoriness of IBD.

This study focused on the protective and repairing effects of the *B. subtilis*-fermented milk on both small intestinal and colonic mucosa in the DSS-induced IBD mouse model and aimed at exploring the action mechanisms of the *B. subtilis*-fermented milk. The results indicated that oral intake of DSS could induce extensive injury of the small intestinal mucosa and the colonic mucosa. In the active phase of the DSS-induced IBD, the major pathological characteristics included necrosis of the epithelium and the ulcers in the mucosa. The damage in the colon was more severe than in the small intestine. In the recovery stage, injury in the small intestine could partially recover automatically, while the ulcers of the colonic mucosa were replaced by inflammatory scars. For the small intestine, in the active phase (DSS-inducing phase), oral intake of the *B. subtilis*-fermented milk could prevent inflammatory injury, and in the recovery phase (after the DSS-inducing phase), the *B. subtilis*-fermented milk could promote the repairing of the injury and then completely reconstruct the microstructure of the mucosa. For the colon, in the active phase, oral intake of the *B. subtilis*-fermented milk could lessen the ulcer of the mucosa, and in the recovery phase, oral intake of the *B. subtilis*-fermented milk could inhibit the formation of the scars in the colonic mucosa and promote the regeneration of the epithelium. These results suggested that *B. subtilis*-fermented milk possessed the double function of both prevention and alleviation for DSS-induced IBD. Meanwhile, the results also showed that oral intake of the *B. subtilis*-fermented milk could induce goblet cells to secrete more mucus which was stained blue *via* Alcian blue. The mucus secreted by the goblet cells was important for the construction of an integrated mucus barrier, which can protect the deep area of the mucosa from the infiltration of the pathogenic bacteria and, thus, inhibit the local inflammatory reaction (Bergstrom and Xia, 2013; Birchenough et al., 2015).

In this research, the action mechanisms of the *B. subtilis*-fermented milk for treatment of DSS-induced IBD were explored. The *B. subtilis*-fermented milk could inhibit the MPO^+^ neutrophil infiltration and the expression of pro-inflammatory cytokine TNF and promote the expression of the anti-inflammatory cytokine IL-10 in the intestinal mucosa, which reduced local inflammatory injury of the intestinal mucosa. It has been reported that TNF was overexpressed in the intestinal mucosa and could lead to apoptosis of the intestinal epithelial cells (IECs) in IBD (He et al., 2012; Wildenberg et al., 2017; Wong et al., 2020). However, anti-inflammatory cytokine IL-10 exerted essential functions to maintain tissue homeostasis during infection and inflammation through restriction of excessive inflammatory responses and promotion of tissue repairing mechanisms (Pan et al., 2013; Koelink et al., 2020). IL-10 could be secreted by both immune cells and IECs (Are et al., 2008; Hyun et al., 2015). IL-10 binds to a specific receptor on IECs and may regulate the contribution of epithelial cells to the inflammatory and immune response in the digestive tract *via* the autosecretion pathway (Denning et al., 2000; Li et al., 2014; Olszak et al., 2014; Aǧaç et al., 2018). Many experimental results indicated that IL-10-deficient mouse has susceptibility to IBD, and overexpression of IL-10 was related to good therapeutic effect of drugs for IBD (Zhou et al., 2004; Engelhardt and Grimbacher, 2014; Keubler et al., 2015). In this study, the results of immunohistochemical staining and western blotting indicated that oral intake of the *B. subtilis*-fermented milk could inhibit the expression of TNF and promote overexpression of IL-10 in the IECs, which might be an important action mechanism of the *B. subtilis* in the treatment of DSS-induced IBD.

The results of IHC and western blotting for the marker of intestinal stem cells (ISCs) Lgr5 and epithelial marker CDX2 indicated that oral intake of the *B. subtilis*-fermented milk could promote epithelial regeneration *via* protection of the ISCs from inflammatory injury and induced proliferation of the ISCs. ISCs can proliferate and differentiate into intestinal epithelial cells and goblet cells (Sato et al., 2011; Sato and Clevers, 2013; Koo and Clevers, 2014; Sugimoto et al., 2018; Harnack et al., 2019). DSS led to the loss of Lgr5^+^ cells; however, *B. subtilis* could increase Lgr5^+^ ISCs and then result in the regeneration of the epithelium lining the intestinal mucosa injured by DSS-induced IBD. In addition, the *B. subtilis*-fermented milk could induce the small intestinal and colonic epithelium to overexpression of the CDX2. CDX2 is an intestinal specific transcription factor located in the nuclei of IECs and modulates a diverse set of cellular behaviors, including cell proliferation and differentiation, and cell adhesion and migration. CDX2 is an essential regulator of intestinal epithelium homeostasis (Coskun, 2014). TNF-α could impair the functions of CDX2 in IBD leading to mucosal injury (Coskun et al., 2012). Inducing the expression of CDX2 by the *B. subtilis*-fermented milk suggested that *B. subtilis* could maintain intestinal epithelium homeostasis *via* its inhibition against the TNF-α or *via* direct action to stimulate the expression of CDX2. Meanwhile, oral intake of the *B. subtilis-*fermented milk could promote expression of Mucin2 in the goblet cells and expression of villin in the epithelial cells. Mucins are the main components of mucus, which is secreted by goblet cells and forms a protective homeostatic barrier between the resident microbiota and the underlying immune cells in the colon (Derrien et al., 2010; Tailford et al., 2015). It has been reported that 5 weeks after birth, Mucin2 knockout animals develop spontaneous colitis and display increased susceptibility to experimental DSS colitis (Capaldo et al., 2017). Plaisancié et al. (2013) reported that a novel bioactive peptide produced from bovine β-casein in yogurts could induce expression of the gel-forming Mucin2 mucin in the human intestinal mucus-producing cells (HT29-MTX) and enhanced the number of goblet cells and Paneth cells along the small intestine. In this study, overexpression of Mucin2 by oral intake of the *B. subtilis-*fermented milk suggested that *B. subtilis* and/or some bioactive peptide produced from bovine β-casein in the fermented milk might play a role in the treatment of DSS-induced IBD *via* the protective function of Mucin2 as well. Villin is an actin regulatory protein expressed in the intestinal epithelium and possesses the epithelial cell-specific anti-apoptotic function (Friederich et al., 1990). The absence of villin predisposes mice to DSS-induced colitis by inducing apoptosis of the IECs (Wang et al., 2008). Inducing the expression of villin suggested that the *B. subtilis*-fermented milk could play the anti-apoptotic role in the treatment of DSS-induced IBD. More importantly, oral intake of the *B. subtilis-*fermented milk could promote the expression of ZO-1 which showed a dotted-line-like localization on the membrane of the IECs. The proteins of tight junction, ZO-1 and ZO-2, can bind directly to F-actin and other cytoskeletal proteins, and these proteins are relevant both to cellular organization and epithelial morphogenesis (Buckley and Turner, 2018; Roy et al., 2018). It has been reported that the expression of ZO-1 was significantly higher in the patients with quiescent UC with mucosa healing compared with those without mucosal healing, and the loss of ZO-1 could increase the permeability of the intestinal epithelium and promote the development of significant intestinal inflammation in animals with DSS colitis (Poritz et al., 2007; Fanning et al., 2012). Peng et al. (2019) reported that the probiotic *B. subtilis CW14* could reduce disruption of the epithelial barrier and toxicity of ochratoxin A to Caco-2 cells *via* improving ZO-1 protein expression. In this study, the high expression level and the dotted-line-like distribution of ZO-1 along the surface of the IECs in the normal group and the DSS + *B. subtilis-*fermented milk group suggested that ZO-1 participated in the construction of the tight junction between the intestinal epithelial cells. These results suggested that *B. subtilis* could promote the reconstruction of the epithelium barrier which prevented pathogenic bacterial invasion and protected the intestinal mucosa from inflammatory injury in DSS-induced IBD.

The inflammatory injury in the intestine was reported to be related with the imbalance of the intestinal flora and reduction of the abundance and diversity of the gut microbiota (Cabré and A Gassull, 2007; Hernández-Chirlaque et al., 2016). As reported by Sjöberg et al. (2017; Mentella et al., 2020) pyrosequencing revealed that the gut microbiota of patients with ulcerative colitis contained fewer operational taxonomic units (OTU) per individual than the controls, and this reduction in richness of the gut microbiota was observed in *Firmicutes*, *Actinobacteria*, *Collinsella*, *Lactobacillus*, and *Bacillus*. In this research, oral intake of the *B. subtilis*-fermented milk increased the species diversity of the normal intestinal microbiota. Also, a decrease in the abundance of *Alistipes*, *Rikenella*, *Barnesiella*, *Macellibacteroides*, and *Lactobacillus* was observed in the DSS-induced IBD models, while the abundance of *Escherichia* and *Bacteroides* increased. According to the LEfSe analysis results, *Clostridiales* and another genus in *Clostridium* (*Clostridium_IV*) were the most significantly increased genus in the DSS group and the DSS + milk group. Some species in the *Clostridium* genus were reported to be opportunistic pathogens associated with intestinal infection. *Clostrodium difficile* was reported to be related with diarrhea (Cabré and A Gassull, 2007). In accordance with our results, it was reported in several researches that the relative abundance of *Bacteroides* was higher in DSS-induced IBD models (Goldenberg et al., 2017). On the other hand, the results suggested that the *B. subtilis*-fermented milk could significantly increase the total species and diversity of the gut microbiota which were reduced by DSS-induced IBD. Meanwhile, the fractions of *Lactobacillae* and *Porphyromonadaceae* in the DSS + *B. subtilis*-fermented milk group were increased to a similar level of the normal group. Oral intake of the *B. subtilis*-fermented milk significantly increased the abundance of *Bacillus*, *Barnesiella*, *Alistipes*, and *Saccharibacteria*, and the total OTU detected was largely increased. Another genus in *Clostridiaceae* (different from the genus detected in other groups) was detected in the DSS + *B. subtilis*-fermented milk group, but the LDA score was lower than the *Clostridiales* genus detected in the DSS group. Meanwhile, the abundance of *Escherichi*a, which was reported to be related with inflammatory reactions, was reduced (Munyaka et al., 2016; Lee et al., 2019). The dramatic increase of *Bacillus* abundance after oral intake of the *B. subtilis*-fermented milk suggested that *B. subtilis* was successfully implanted in the intestine. Among the species increased through *B. subtilis* intake, *Barnesiella* and *Alistipes* have been approved as beneficial to intestinal health. *Alistipes* was reported to reduce inflammatory reaction in the intestine and regulate lipid metabolism (Dziarski et al., 2016). Also, *Barnesiella* could facilitate cyclophosphamide, which possesses anticancer activity (Daillère et al., 2016). Besides, the abundance of short-chain fatty acid (SCFA)-producing *Ruminococcus* was significantly higher in the DSS + *B. subtilis*-fermented milk group than in the other groups, and it was reported that the absence of *Ruminococcus* was related with CD (El Mouzan et al., 2018; Wang et al., 2018). According to the results in this study, oral intake of *B. subtilis*-fermented milk could efficiently increase the gut flora diversity of the intestinal microbiota and restore the balance of gut flora which was disturbed by DSS. *B. subtilis* in the fermented milk might play an important role in the prevention and alleviation of IBD, *via* regulation of the intestinal flora.

In the current research, the exact molecular mechanism of the therapeutic effect of *B. subtilis*-fermented milk on IBD remains uncovered in this research and needs future research. It was reported recently that the metabolites from *B. subtilis*, including surfactin A and poly-γ-glutamic acid, could attenuate the symptoms of IBD animal models (Davaatseren et al., 2013; Yan et al., 2020). Besides, different bacterial species including *Bacteroides thetaiotaomicron* and *Lactobacillus johnsonii* could modulate the intestinal inflammation *via* enzymatic hydrolysis, and enzymes from *B. subtilis* might also contribute to its prevention and alleviation effect on IBD (Charlet et al., 2020). Nattokinase from *B. subtilis* was reported to inhibit inflammation and restrain oxidative stress in mice (Wu et al., 2020), and chitinase from *B. subtilis* showed antifungal activity through degradation of the cell wall (Manjula and Podile, 2005), which might contribute to the regulation of the intestinal flora.

## Conclusion

This research focused on the prevention and alleviation effects of the *B. subtilis*-fermented milk on the DSS-induced IBD and explored the action mechanisms, including inhibition of inflammation, promotion of reconstruction of intestinal mucosal barrier, and regulation of the intestinal flora. It was demonstrated that oral intake of the *B. subtilis*-fermented milk could reduce inflammatory injury of both the small intestinal and colonic mucosa and promote epithelial regeneration and reconstruction of the intestinal mucosa barrier. An oral supplement of *B. subtilis* could increase the total species and diversity of the bowel microbiota and regulate the gut flora balance which was disturbed by DSS-induced IBD. The results indicated that oral intake of the *B. subtilis*-fermented milk could prevent and cure the DSS-induced IBD in mice. The *B. subtilis*-fermented milk would be a potential novel functional food for application in the therapy of IBD. However, it remains to be further explored what bioactive ingredients (the probiotic *B. subtilis*, its metabolites, or these cooperative factors) in the *B. subtilis-*fermented milk possessed the functions for the prevention and alleviation of IBD. In addition, the molecular signaling pathway related to the function of *B. subtilis* in the treatment of IBD is worthy of further study.

## Data Availability Statement

The datasets presented in this study can be found in online repositories. The names of the repository/repositories and accession number(s) can be found below: NCBI BioProject, accession no: PRJNA679432.

## Ethics Statement

The animal study was reviewed and approved by the Ethics Committee of Jiangsu University.

## Author Contributions

XZ: data collection, data analysis, and initial manuscript drafting. YT: study design, data analysis, and initial manuscript drafting. XL: data analysis and manuscript reviewing and revision. JW and YW: data collection and data analysis. RY: study design, data analysis, and manuscript reviewing and revision. All authors contributed to the article and approved the submitted version.

## Conflict of Interest

The authors declare that the research was conducted in the absence of any commercial or financial relationships that could be construed as a potential conflict of interest.
